# Editorial: Retrieving meaningful patterns from big biomedical data with machine learning approaches

**DOI:** 10.3389/fgene.2023.1177996

**Published:** 2023-03-17

**Authors:** Yun Zheng, Zexuan Zhu

**Affiliations:** ^1^ College of Landscape and Horticulture, Yunnan Agricultural University, Kunming, Yunnan, China; ^2^ National Engineering Laboratory for Big Data System Computing Technology, College of Computer Science and Software Engineering, Shenzhen University, Shenzhen, Guangdong, China

**Keywords:** big biomedical data, machine learning, classification, regression, prognosis, software tool

In recent years, a huge amount of biomedical data has been generated by high throughput sequencing facilities. These data include, but not limited to, genomic sequences, transcriptome profiles, non-coding RNA profiles, epigenetics profiles, and single cell RNA-Seq profiles. These data sets are normally large and noisy. Therefore, careful analysis with machine learning methods is often needed to extract meaningful patterns from these data. Machine learning problems were normally grouped into classification and regression problems, whose variables under consideration were categorical (qualitative) and numerical (quantitative), respectively (James et al., 2013).

This Research Topic of Frontiers in Genetics features a Research Topic of three articles for identifying meaningful molecular patterns from clinical samples and an article that introduces a web-based tool for visualization of multi-omics microbial data sets.

Early detection of cancer is critical for better outcome and lower mortality (Siegel et al., 2020). The task is very challenging since clinical tumor samples of some cancers are unaccessible. It is therefore very valuable if early detection of cancer could be reliably conducted with accessible clinical samples, such as blood samples. However, the sensitivities of existing methods (Cohen et al., 2018; Liu et al., 2020) are not satisfactory. Qi et al. collected 75 whole-blood transcriptome profiles from 45 patients with various non-blood cancers (breast, esophagus, stomach, thyroid, rectum, colon, and uterus) and 30 normal controls. They first identified 900 differentially expressed genes (DEGs) from a training set with 53 samples (Qi et al.). Then, the support vector machine (SVM) algorithm was used to build models based on these 900 DEGs, 120 very long intergenic non-coding RNAs (vlincRNAs), and 780 non-vlincRNA genes, respectively. Qi et al. showed that these SVM-based models were accurate for pan-cancer detection on the independent testing data with 22 samples. They also demonstrated that vlincRNAs had superior performance when compared to protein-coding mRNAs (Qi et al.).

Zhong et al. constructed a hypoxia-related prognosis model, namely, HPM based on gene signatures and machine leaning methods to predict the survival of acute myeloid leukemia patients. The proposed HPM and the derivative models with clinical risk factors were validated with various experimental studies including Kaplan-Meier survival analysis, time-dependent ROC analysis, clinical characteristics analysis, and hypoxia-related immune and metabolic alterations (Zhong et al.). The HPM and its derivative models were able to effectively predict the survival of AML patients, which might improve risk classification (Zhong et al.).

Based on cuproptosis-related genes reported in (Tsvetkov et al., 2022), Cai et al. identified 956 cuproptosis-related lncRNAs (CRLs). Then, univariate Cox regression was utilized to identify 69 CRLs in the training group and multivariate Cox regression was used to identify 3 CRLs as independent prognostic factors (Cai et al.). And the regression model based on these three CRLs could accurately predict the prognosis of bladder cancer (BC) patients (Cai et al.). They also showed that high-risk BC patients benefited more from immunotherapy and had stronger immune responses, and their overall survival was better (Cai et al.).

Li et al. developed MicrobioSee, a web-based toolkit for visualizing multi-omics data sets of microorganisms. MicrobioSee provided non-expert users friendly interfaces for 17 different analysis tasks of major omics data of microorganisms (Li et al.).

In summary, the work in this Research Topic provided some practical examples of employing machine learning methods in multi-omics profiles of diseases. Interesting patterns were identified in these studies in the contexts of classification and regression problems.
